# Supplementary material for: Inhibiting the system xC−/glutathione axis selectively targets cancers with mutant-p53 accumulation
Source: Nat Commun. 2017 Mar 28;8:14844. doi: 10.1038/ncomms14844 (PMC5379068; doi:10.1038/ncomms14844)
Supplement: Supplementary Information — Supplementary Figures, Supplementary Tables and Supplementary References. [file ncomms14844-s1.pdf]

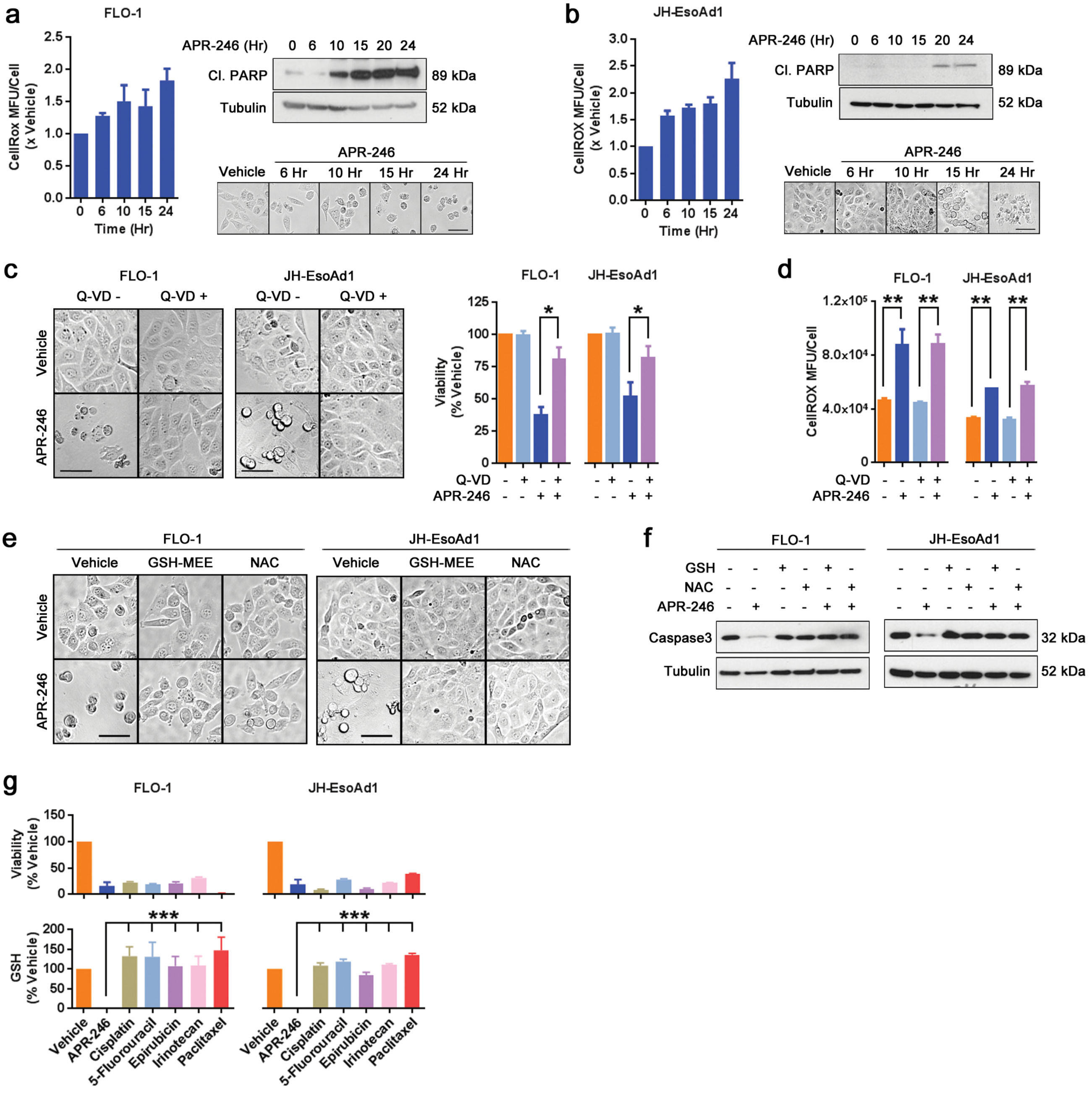

Supplementary Figure 1. Related to Figure 1. Glutathione depletion and ROS induction is central to the anti-tumour activity of APR-246. For all experiments, FLO-1 and JH-EsoAd1 cells were treated with 25 and 40 μM of APR-246 respectively. (**a-b**), ROS levels (left), western blot (top right) and bright-field microscopy (bottom right) of FLO-1 (**a**) and JH-EsoAd1 (**b**) cells post APR-246 treatment. Scale bar=100 μm. (**c**) Bright-field microscopy (left) and viability (right) of FLO-1 and JH-EsoAd1 cells following treatment with the pan-caspase inhibitor Q-VD (25 μM) and/or APR-246. Cells were pre-incubated with Q-VD for 1 hr prior to APR-246. Bright-field images were captured at 24 hr and viability assayed at 72 hr post APR-246. Scale bar=100 μm, n=2. (**d**) Reactive oxygen species (ROS) detected using CellROX at 10 hr post treatment conditions detailed in (**c**). Mean fluorescence unit (MFU), n=2. (**e**) Bright-field microscopy of FLO-1 and JH-EsoAd1 cells captured at 24 hr post treatment with APR-246 and/or 5 mM glutathione-monoethyl ester (GSH-MEE) or N-acetyl cysteine (NAC). Scale bar=100 μm. (**f**) Western blot of cells harvested at 15 hr post treatment conditions detailed in (**e**). n=3. (**g**) Viability (top) and GSH (bottom) assayed at 96 and 10 hrs respectively post treatment with APR-246 (FLO-1: 25 μM, JH-EsoAd1: 40 μM), cisplatin (FLO-1: 10 μM, JH-EsoAd1: 10 μM), 5-fluorouracil (FLO-1: 10 μM, JH-EsoAd1: 10 μM), epirubicin (FLO-1: 100 nM, JH-EsoAd1: 500 nM) or irinotecan (FLO-1: 10 μM, JH-EsoAd1: 50 μM) or paclitaxel (FLO-1: 50 nM, JH-EsoAd1: 50 nM). n=3. One-way ANOVA with Dunnett's multiple comparison posttest (**c, g**), or with Tukey's multiple comparison posttest (**d**). Error bars=SEM, *p<0.05, **p<0.01, ***p<0.001.

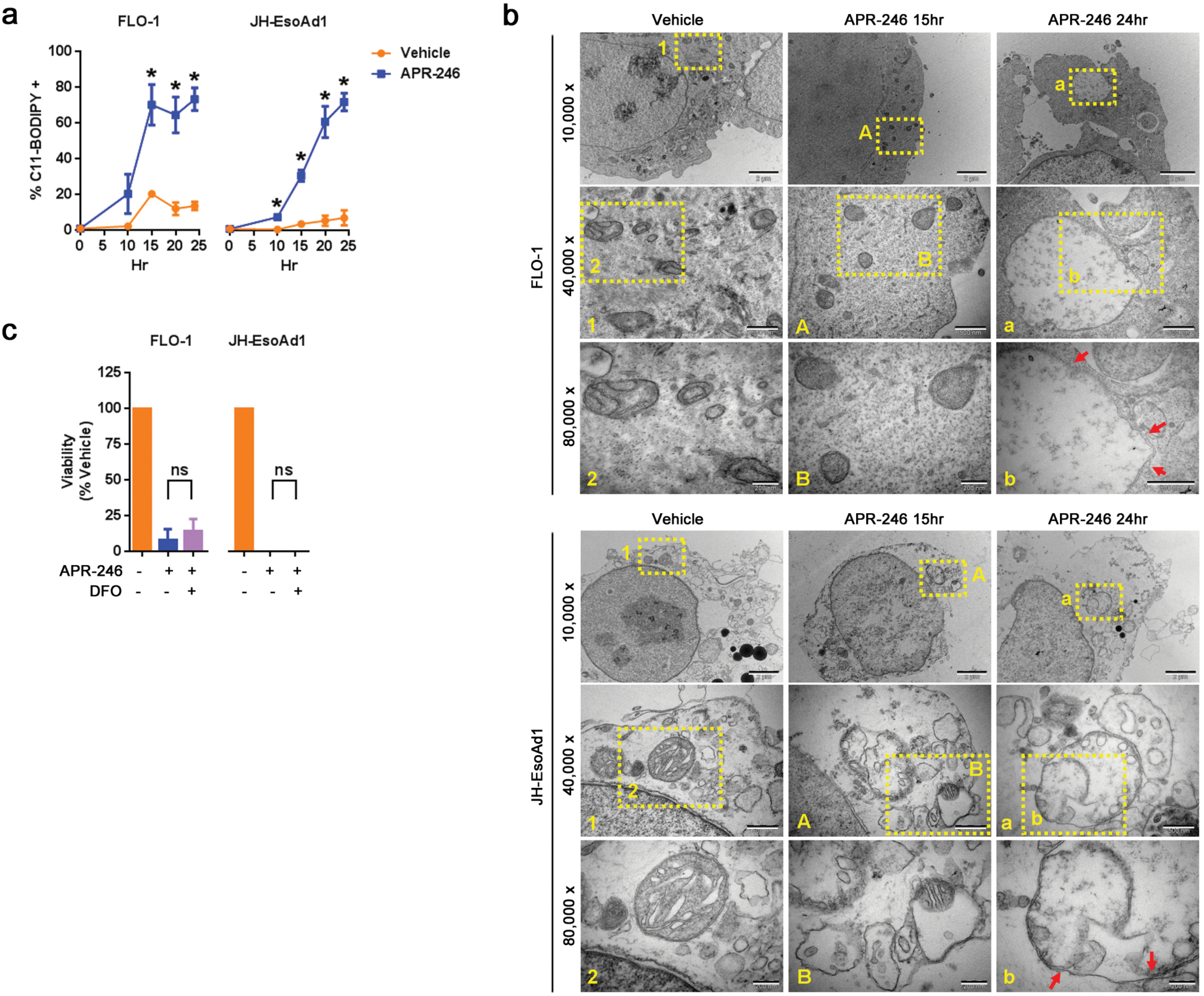

Supplementary Figure 2. Related to Figure 2. APR-246 triggers lipid peroxidative cell death through depleting glutathione. (**a**) Lipid peroxidation detected using C11-BODIPY dye post APR-246 treatment. n=3. (**b**) Transmission electron microscopy of FLO-1 and JH-EsoAd1 cells treated with APR-246. Red arrows: mitochondrial membrane rupture. A minimum of 10 cells were examined. Scale bar: 10,000x=2 µm, 40,000x=500 nm, 80,000x=200 nm. (**c**) Viability assayed at 96 hr post treatment with APR-246 and/or deferoxamine (DFO, 100 µM). n=3. Unpaired t-test (**a**) and one-way ANOVA with Dunnett's multiple comparison posttest (**c**). Error bars=SEM, *p<0.05, not significant (ns).

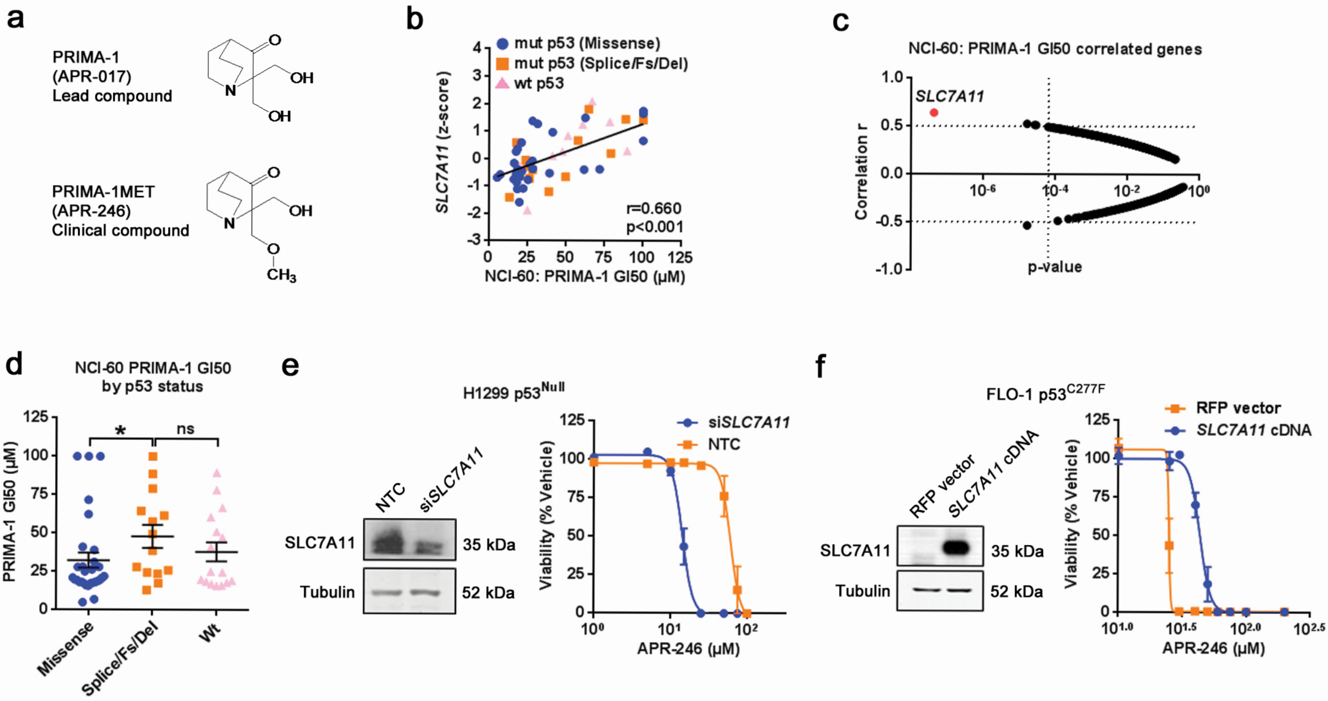

Supplementary Figure 3. Related to Figure 3. *SLC7A11* expression predicts and modulates tumour sensitivity to APR-246. (**a**) Structural formula of APR-246 and its lead compound PRIMA-1 (APR-017). (**b**) Pearson's correlation between *SLC7A11* mRNA levels with PRIMA-1 GI50 across the NCI-60 cancer cell line panel. Each point represents an individual cell line. The NCI-60 is a panel of 60 diverse human cancer cell lines used by the National Cancer Institute for therapeutic development. (**c**) Plot of 925 genes whose expression correlated with PRIMA-1 GI50 with a $|r|>0.3$ as determined from 5 NCI-60 microarray datasets. Horizontal and vertical dotted lines indicate r=0.5 and Bonferroni corrected p-value=5.4×10^{-5} respectively. (**d**) PRIMA-1 GI50 of the NCI-60 cancer cell lines grouped according to their *TP53* status. Unpaired t-test. Error bars=SEM, *$p<0.05$, not significant (ns). (**e**) *SLC7A11* knockdown in $p53^{Null}$ H1299 cells. APR-246 was applied 2 days post transfection of *SLC7A11* and non-targeting control (NTC) siRNA with viability measured at 96 hr post APR-246 (right). Knockdown was confirmed by western blot (left) 3 days post transfection. (**f**) *SLC7A11* and red fluorescent protein (RFP) were overexpressed in mut-p53 FLO-1 cells. This was confirmed by western blot (left). Cell viability was measured 96 hr post APR-246 (right).

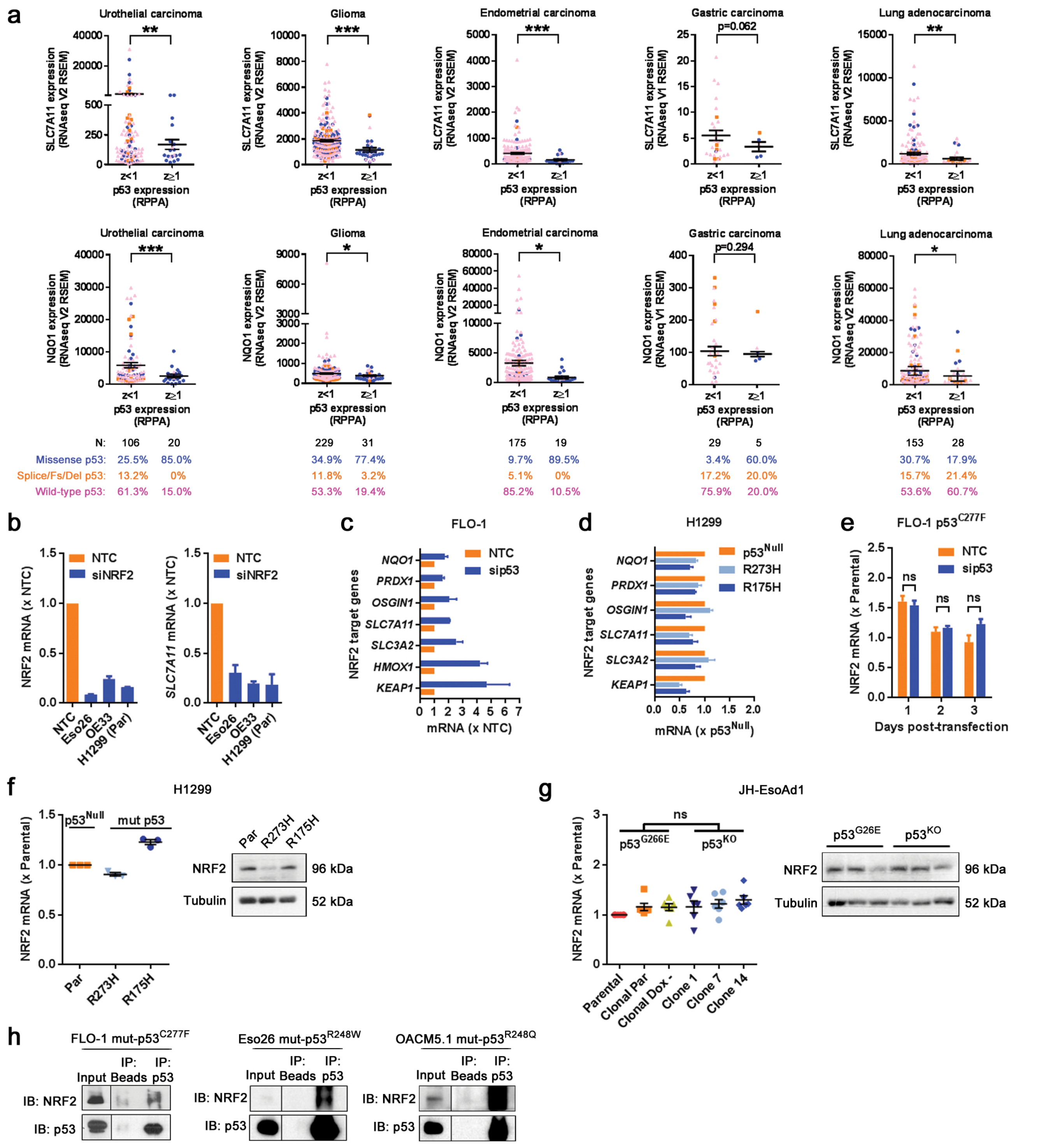

Supplementary Figure 4. Related to Figure 4. Accumulated mut-p53 entraps NRF2 and represses *SLC7A11* expression. (**a**) TCGA dataset analysis: correlation between p53 protein levels (Reverse phase protein array - RPPA, z≥1: high expression, z<1: low expression) with *SLC7A11* (top) and *NQO1* (bottom) mRNA expression (RNAseq) in multiple tumour types. (**b**) mRNA expression of NRF2 (left) and *SLC7A11* (right) 72 hr following siNRF2 or non-targeting control (NTC) siRNA transfection in Eso26, OE33 and H1299 parental (par) $p53^{Null}$ cells. (**c-d**) mRNA expression of selected NRF2 anti-oxidative stress genes following mut-p53 knockdown (**c**, 72 hr post sip53 or NTC siRNA transfection) and overexpression (**d**) in FLO-1 and H1299 cells respectively. (**e**) NRF2 mRNA levels 72 hr following mut-p53 knockdown in FLO-1 cells. (**f-g**) NRF2 mRNA (left) and protein (right) levels following mut-p53 overexpression (**f**) and knockout (**g**) in H1299 and JH-EsoAd1 cells respectively. (**h**) Immunoprecipitation (IP) and immunoblot (IB) of mut-p53 with NRF2 in FLO-1, Eso26 and OACM5.1 cells under basal growth conditions. Unpaired t-test (**a, e, g**). Error bars=SEM, **p<0.01, ***p<0.001, not significant (ns). n=3 for all in vitro studies except (**g**) n=6.

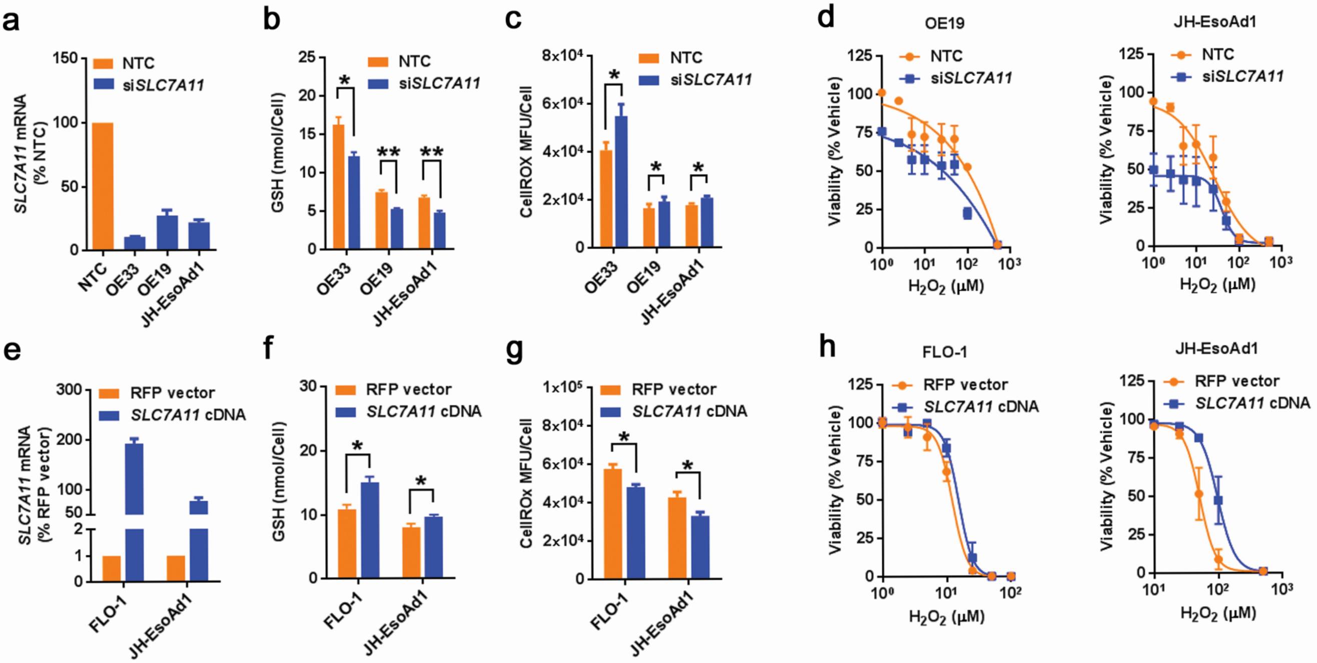

Supplementary Figure 5. Related to Figure 5. Mut-p53 accumulation sensitises cancer cells to oxidative stress. (**a-c**) *SLC7A11* mRNA levels (**a**), glutathione (GSH) levels (**b**), and reactive oxygen species (ROS) detected using CellROX (**c**) 48 hr following si*SLC7A11* and non-targeting control (NTC) transfection in OE33, OE19 and JH-EsoAd1 cells. Mean fluorescence unit (MFU). (**d**) Sensitivity of OE19 (left) and JH-EsoAd1 (right) cells to H_2O_2 following *SLC7A11* knockdown. H_2O_2 was applied 48 hrs post transfection of siRNA and cell viability assayed at 96 hr post H_2O_2. (**e-h**) *SLC7A11* mRNA levels (**e**), GSH levels (**f**), endogenous ROS detected using CellROX (**g**), and sensitivity to H_2O_2 (**h**) following overexpression of *SLC7A11* or red fluorescence protein (RFP) control cDNA in FLO-1 and JH-EsoAd1 cells. Cell viability was assayed 96 hr post H_2O_2. Unpaired t-test (**b, c, f, g**). Error bars=SEM, *p<0.05, **p<0.01. n=3 for all studies except (**c, d, g**) n=4.

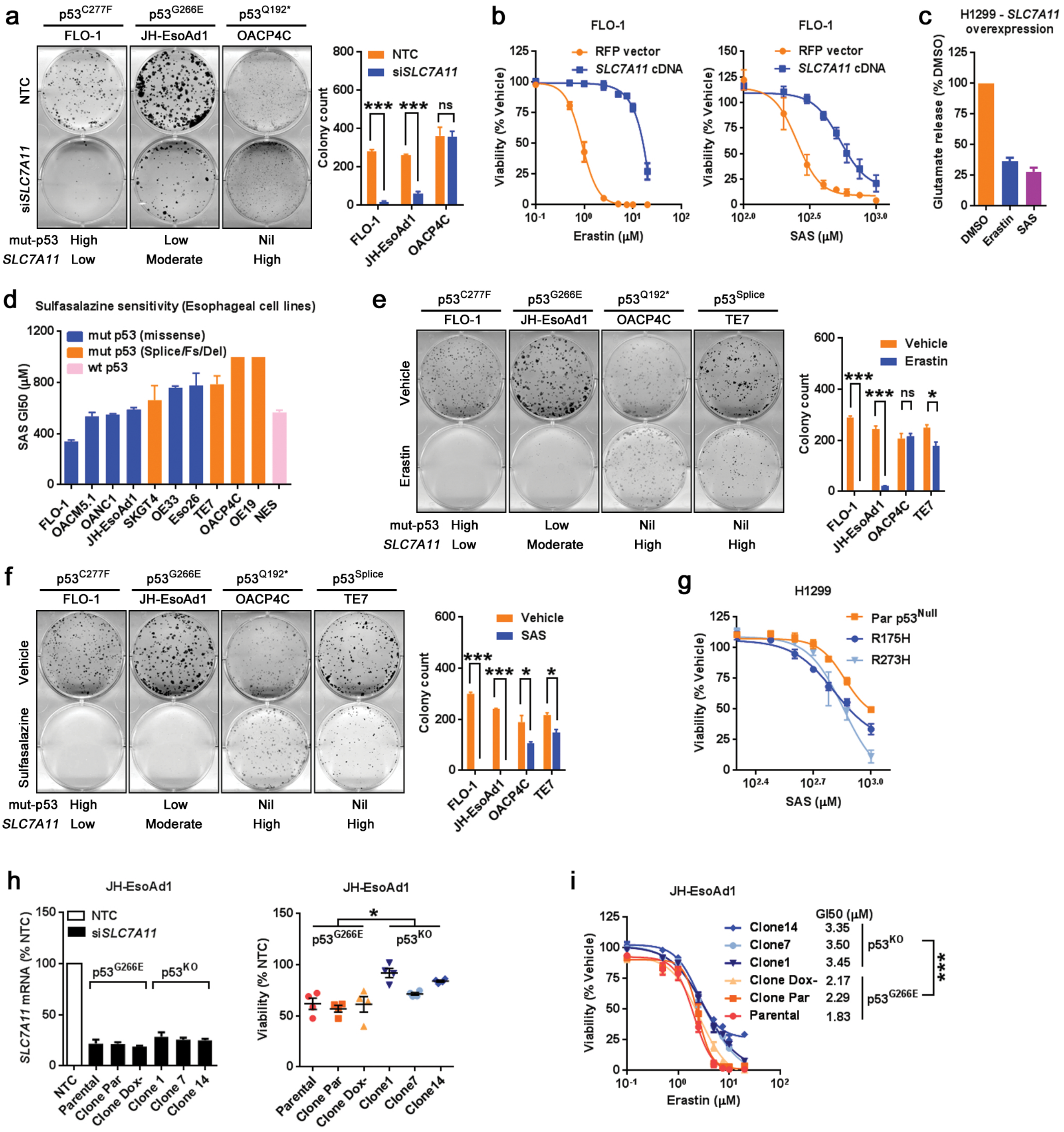

Supplementary Figure 6. Related to Figure 6. System x_c^- inhibition selectively targets cancer cells with mut-p53 accumulation. (**a**) Clonogenic assay of FLO-1, JH-EsoAd1 and OACP4C cells with differing mut-p53 and *SLC7A11* expression following si*SLC7A11* and non-targeting control (NTC) transfection. Representative photograph (left) and colony count (right). (**b**) Cell viability measured at 96 hr following erastin (left) and sulfasalazine (SAS, right) treatment in FLO-1 cells with either *SLC7A11* or red fluorescence protein (RFP) overexpression. (**c**) System x_c^- activity as assayed by glutamate release from H1299 cells overexpressing *SLC7A11*, treated with either vehicle (DMSO), erastin (40 µM) or SAS (1 mM) for 3 hr in the presence of 600 µM of L-cystine. (**d**) SAS sensitivity (GI50) in oesophageal cell lines. (**e-f**) Clonogenic assay of FLO-1, JH-EsoAd1, OACP4C and TE7 cells with differing mut-p53 and *SLC7A11* expression following 2.5 µM erastin (**e**) and 500 µM SAS (**f**) treatment. Representative photograph (left) and colony count (right). (**g**) Cell viability measured at 96 hr following SAS treatment in parental (Par) $p53^{Null}$ and mut-p53 overexpressing H1299 cells. (**h**) *SLC7A11* knockdown in $p53^{G266E}$ and $p53^{KO}$ JH-EsoAd1 cells. *SLC7A11* mRNA (left) and viability (right) measured at 48 and 96 hr post siRNA transfection respectively. (**i**) Viability at 96 hr post erastin treatment in $p53^{G266E}$ and $p53^{KO}$ JH-EsoAd1 cells. Unpaired t-test (**a, e, f, h, i**). Error bars=SEM, *p<0.05, ***p<0.001, non-significant (ns). n=3 for all studies except (**c**) n=2 and (**h**) n=4.

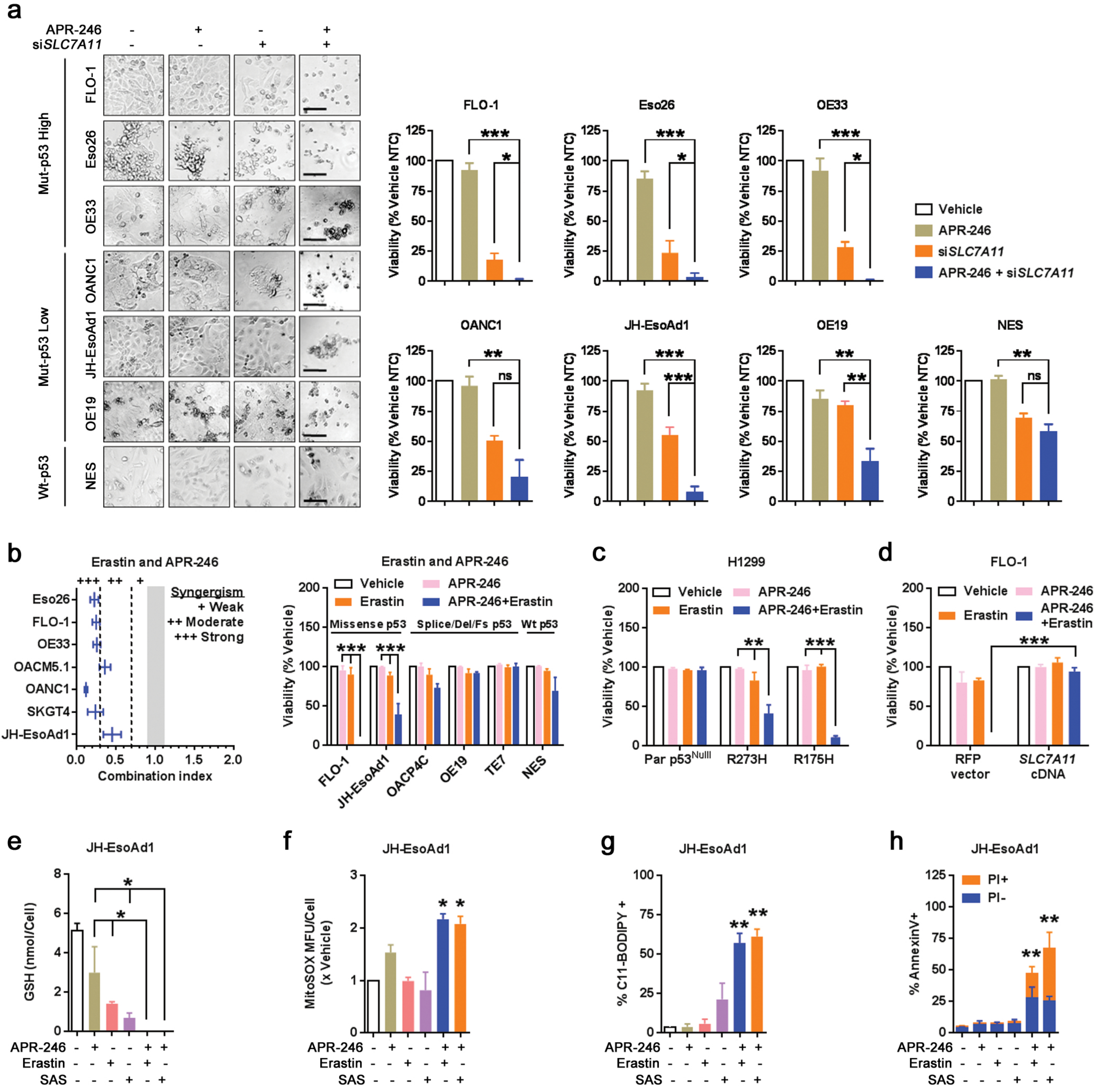

Supplementary Figure 7. Related to Figure 7. System x_c^- antagonists synergise with APR-246 to inhibit mut-p53 cancer cells. (**a**) Bright-field microscopy (left) and viability analysis (right) 96 hr post 10 µM APR-246 treatment in cells transfected with either non-targeting control (NTC) or si*SLC7A11*. Cells were transfected with siRNA 48 hr prior to APR-246. Scale bar=100 µm. (**b**) Combination index (CI) plot (left) and viability analysis (right) of cells treated with APR-246 and erastin for 96 hr. For CI analysis, cells were treated with a range of drug doses. Synergistic interaction was quantified using CalcuSyn v2, where a CI<0.9: synergism, CI>1.1: antagonism and 0.9≤CI≤1.1 (Grey area): additive effect. For single dose viability analysis, cells were treated with 7.5 µM APR-246 and/or 0.5 µM erastin. (**c**) Viability at 96 hr post treatment with 10 µM APR-246 and/or 1 µM erastin in parental (Par) $p53^{Null}$ and mut-p53 overexpressing H1299 cells. (**d**) Viability at 96 hr post treatment with 7.5 µM APR-246 and/or 0.5 µM erastin in FLO-1 cells with either *SLC7A11* or red fluorescence protein (RFP) overexpression. (**e-h**) GSH (**e**), MitoSOX (**f**), C11-BODIPY (**g**) and AnnexinV/PI (**h**) analysis of JH-EsoAd1 cells treated with 7.5 µM APR-246 and/or 0.5 µM erastin or 200 µM sulfasalazine (SAS). GSH, MitoSOX, C11-BODIPY and AnnexinV/PI were assayed at 15, 24, 24, and 48 hr post treatment respectively. Mean fluorescence unit (MFU). For (**b** right, **c-h**) the dose of APR-246, erastin or SAS was deliberately chosen to have low cytotoxicity on its own to highlight the combinatory effect. One-way ANOVA with Dunnett's multiple comparison posttest (**a, b** right, **c, e-h**), unpaired t-test (**d**). Error bars=SEM, *p<0.05, **p<0.01, ***p<0.001. n=3 for all studies.

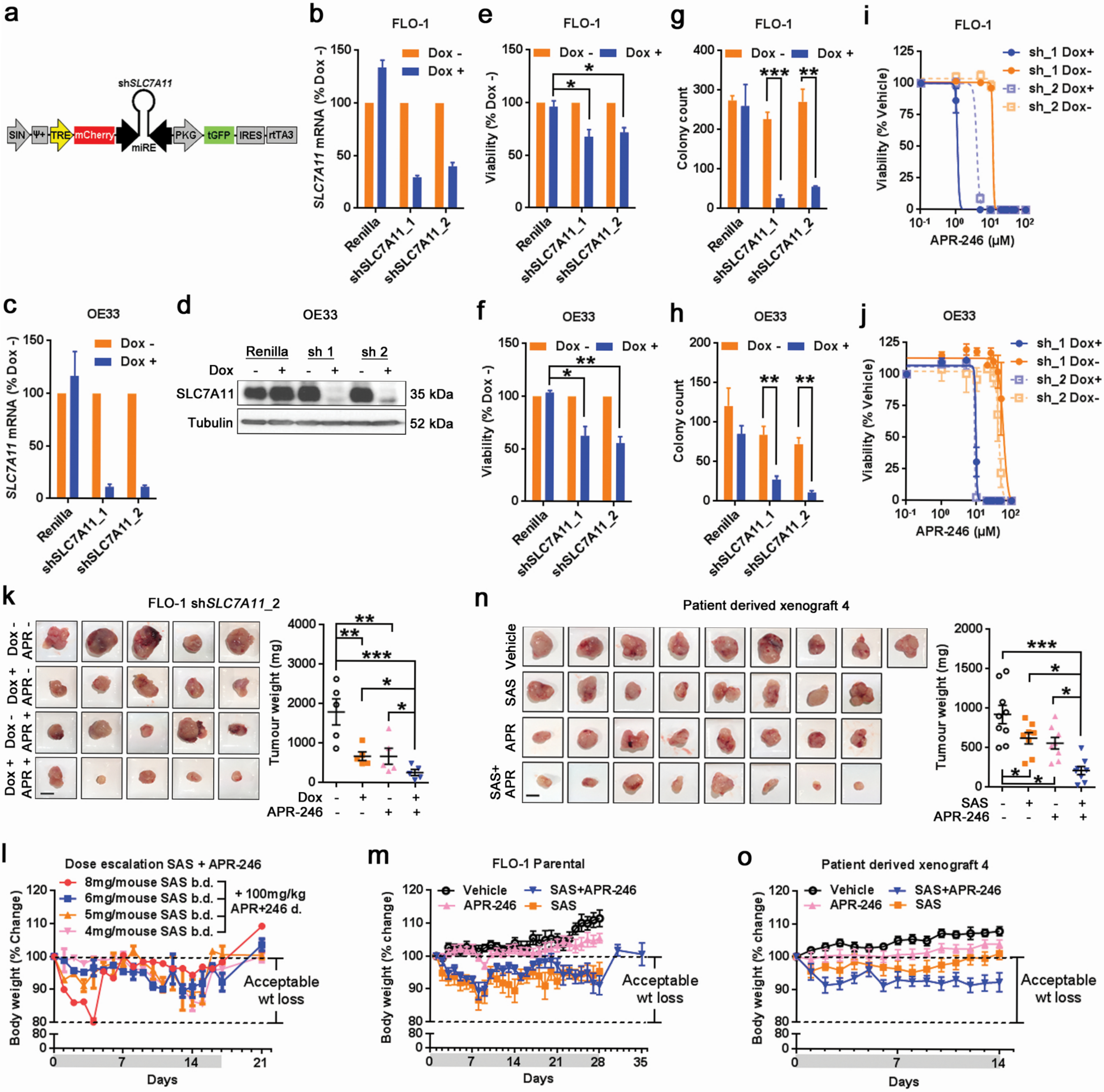

Supplementary Figure 8. Related to Figure 8. System x_C^- antagonist synergise with APR-246 *in vivo*. (**a**) Diagram of the LT3GECIR lentiviral doxycycline (dox)-inducible expression vector containing the optimised miR-E backbone. The mCherry and turbo-GFP reporters are respectively used to indicate transduction and induction. *SLC7A11*-specific shRNAs and shRenilla control shRNA were cloned into this system and transduced into FLO-1 and OE33 cells. (**b-d**) *SLC7A11* mRNA levels in FLO-1 (**b**) and OE33 (**c**) cells, and SLC7A11 protein levels in OE33 cells (**d**) at 72 hr post dox (2 μg ml^{-1}) induction *in vitro*. (**e-f**) Cell viability measured at 96 hr following dox induction in FLO-1 (**e**) and OE33 (**f**) cells. (**g-h**) Clonogenic assay following dox induction in FLO-1 (**g**) and OE33 (**h**) cells. (**i-j**) Cell viability measured at 96 hr following APR-246 treatment in FLO-1 (**i**) and OE33 (**j**) cells. Dox was applied 72 hr prior to APR-246. (**k**) FLO-1 sh*SLC7A11*_2 tumour photographs (left) and weights (right) at experimental endpoint. Scale bar=1 cm. (**l**) Percentage change in body weight as compared with baseline (t=0 days) in a dose escalation experiment of sulfasalazine (SAS; 4, 5, 6 and 8 mg per mouse, twice daily (b.d.)) in combination with APR-246 (100 mg kg^{-1}, daily (d.)) in non-tumour bearing mice. n=2 per group. (**m**) Percentage change in body weight as compared with baseline (t=0 days) following treatment with vehicle (0.9% saline, daily, n=7), APR-246 (100 mg kg^{-1}, daily, n=7), SAS (6 mg per mouse twice daily, n=6) or combination of these doses of SAS and APR-246 (n=6) in mice bearing FLO-1 xenografts. (**n**) Patient derived xenograft 4 (PDX4) tumour photographs (left) and weights (right) at experimental endpoint. Scale bar=1 cm. (**o**) Percentage change in body weight as compared with baseline (t=0 days) following treatment with the same regimen as (**m**) in mice bearing PDX4 tumours (n=9 for vehicle, n=8 for all other groups). For (**l, m, o**), ethically acceptable weight loss is defined by the Peter MacCallum Cancer Centre Animal Experimentation Ethics Committee as less than 20% compared to pre-treatment body weight (within the dotted lines). Grey shading indicates treatment duration. One-way ANOVA with Dunnett's (**e, f**) or Tukey's (**k, n**) multiple comparison posttest, unpaired t-test (**g, h**). Error bars=SEM, *$p<0.05$, **$p<0.01$, ***$p<0.001$. n=3 for all *in vitro* studies.

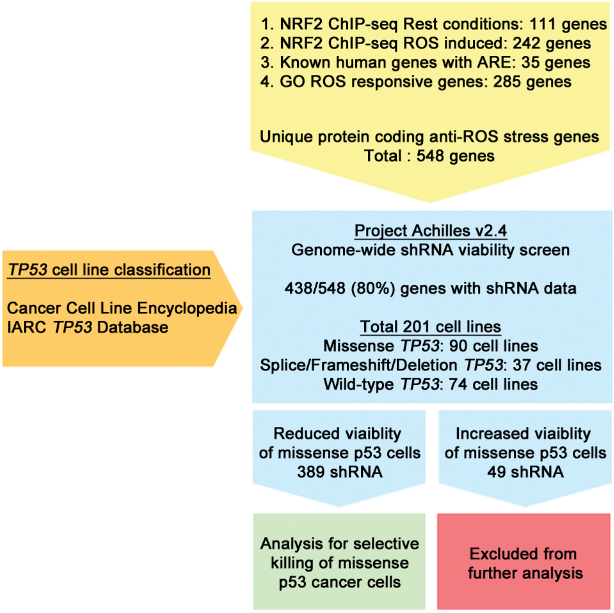

Supplementary Figure 9. Workflow of multi-omics analysis of the Broad Institute's Project Achilles v2.4 dataset. Project Achilles v2.4 is a genome-wide shRNA viability screen in 201 cancer cell lines. These cell lines have different p53 statuses. **Orange box**, sequencing data from the Cancer Cell Line Encyclopedia and the IARC TP53 database was used to categorise the 201 cancer cell lines in Project Achilles v2.4 according to their p53 status. **Yellow box**, a comprehensive list of 548 anti-oxidative stress genes were compiled from several sources: 1) Chromatin immunoprecipitation sequencing (ChIP-seq) of NRF2 under basal and ROS induced conditions as identified by Chorley et al[1], 2) known human genes with functionally validated Anti-oxidant Responsive Elements (ARE) as reviewed by Chorley et al[1], and 3) Gene Ontology (GO) designated ROS responsive genes[2]. **Blue box**, of the 548 anti-oxidative stress genes shortlisted, 438 were available for interrogation using Project Achilles v2.4. Of these, knockdown of 389 genes were found to reduce the viability of missense mut-p53 cancer cells. **Green box**, these 389 candidate genes were selected for further analysis to determine which when inhibited would preferentially reduce the viability of missense mut-p53 cancer cells compared to non-missense p53 cells. **Red box**, the remaining 49 genes were excluded from further analysis as they increased the viability of missense p53 cells when inhibited.

Supplementary Figure. 1a-b

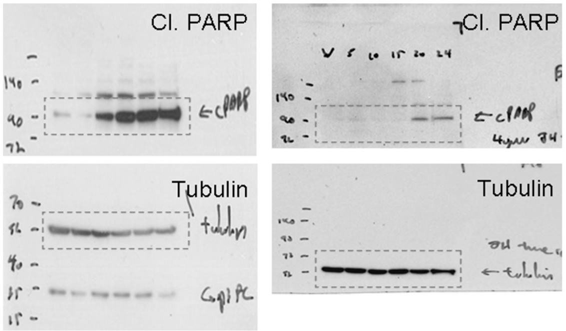

Supplementary Figure. 1f

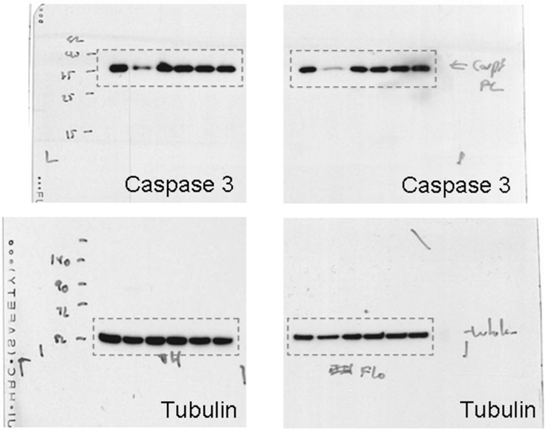

Figure. 3c

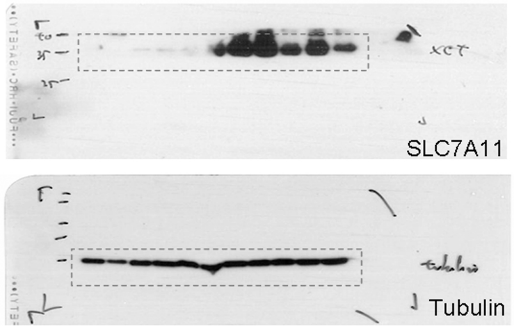

Figure. 3g and Supplementary Figure. 3e

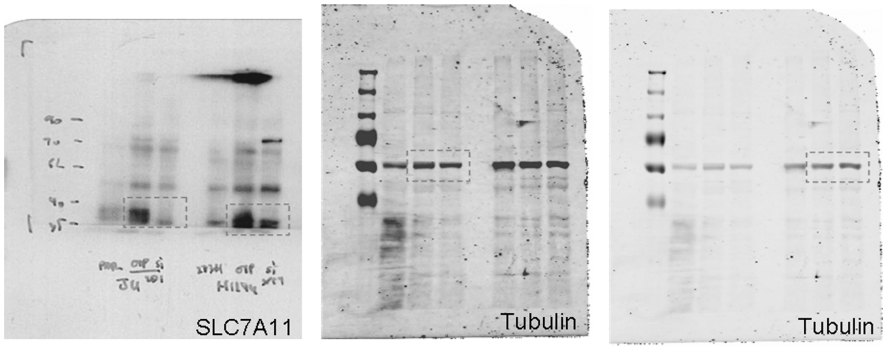

Figure. 3h and Supplementary Figure. 3f

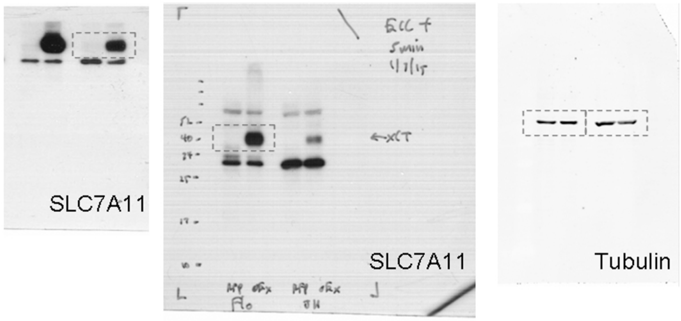

Figure 3i

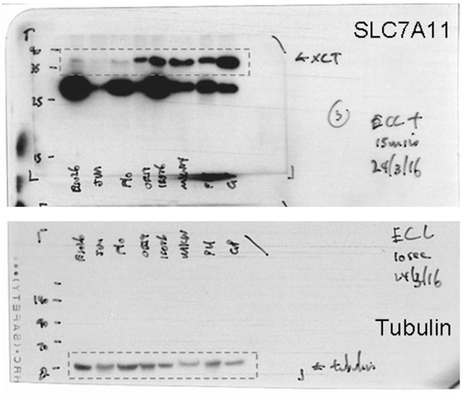

Supplementary Figure 10. Extended western blots. The title of each western blot corresponds to its cropped version within the paper.

Figure. 4a

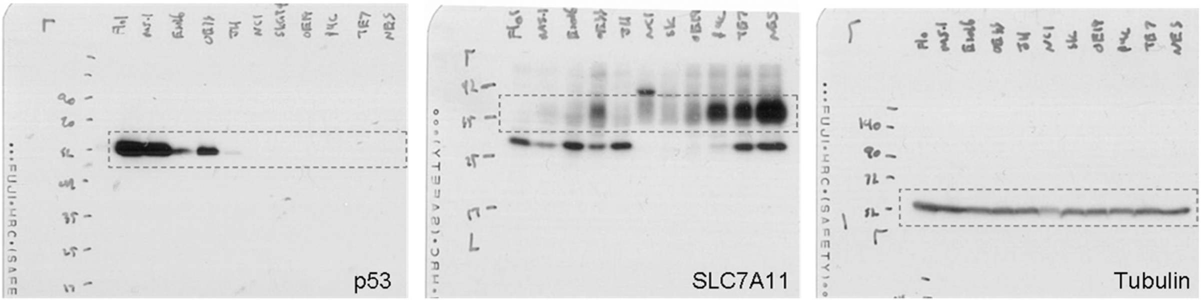

Figure. 4c

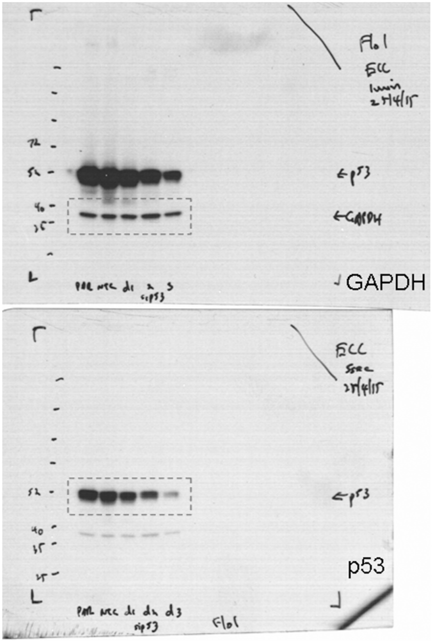

Figure. 4d

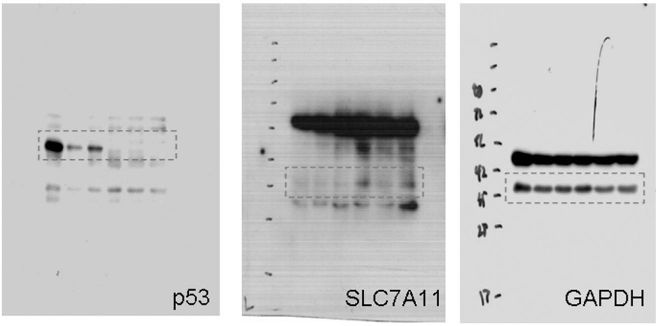

Figure. 4e

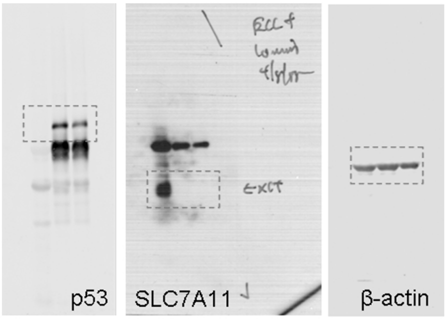

Supplementary Figure. 4f-g

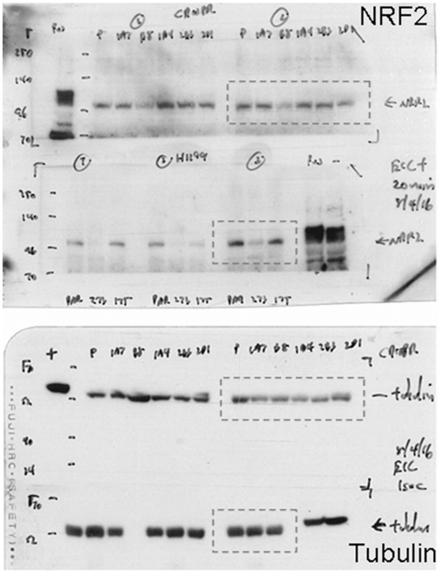

Supplementary Figure 10. Extended western blots. The title of each western blot corresponds to its cropped version within the paper.

Figure. 4f and Supplementary Figure. 4h

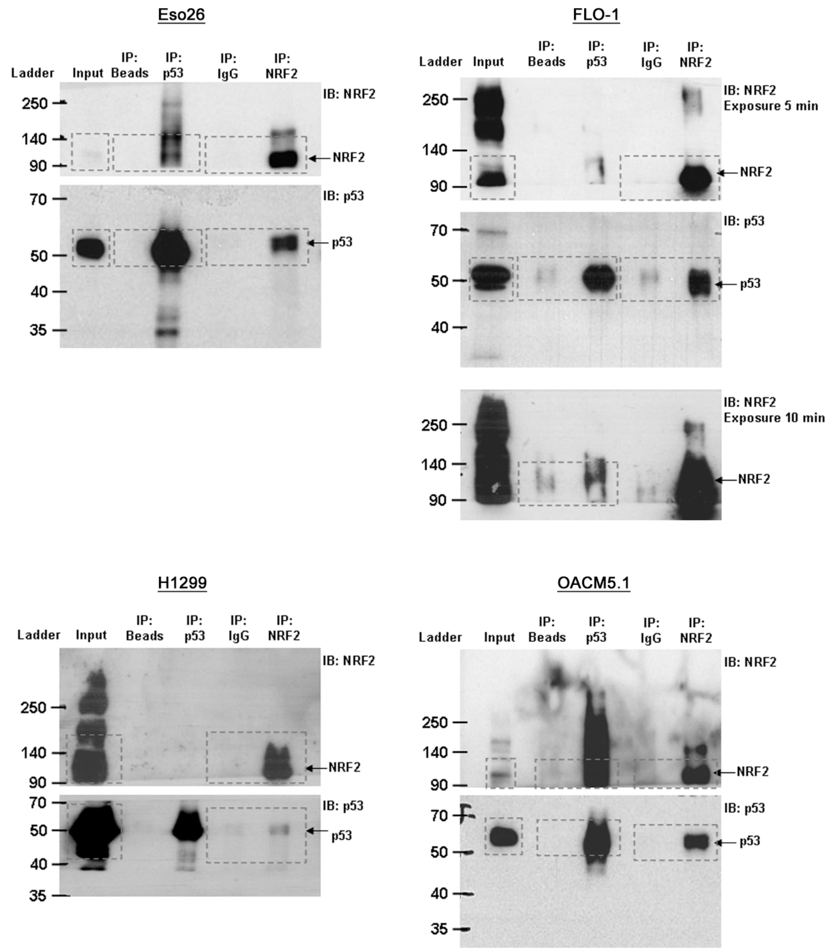

Figure. 8a

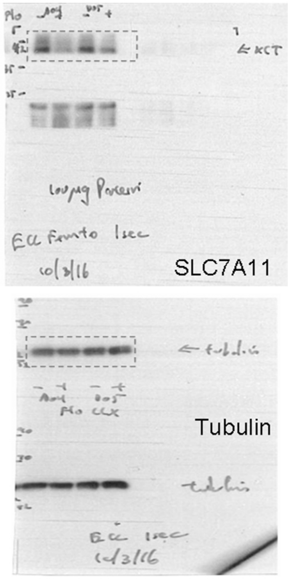

Supplementary Figure. 8d

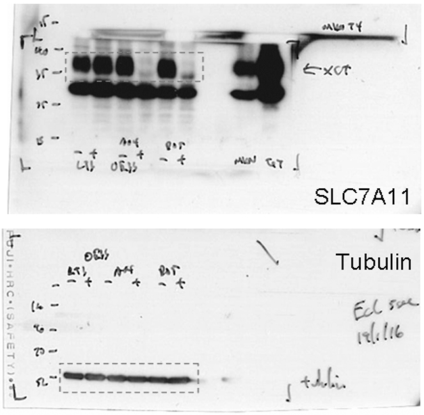

Figure. 8h

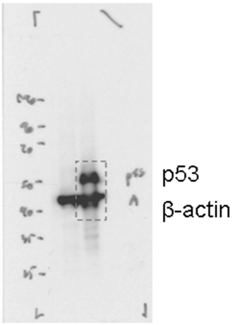

Supplementary Figure 10. Extended western blots. The title of each western blot corresponds to its cropped version within the paper.

Supplementary Tables

Supplementary Table 1. p53 status of cell lines and PDXs and their sensitivity to APR-246

Cell lines	Origin	*TP53* gene mutation	Protein phenotype	p53 protein domain	Functional phenotype	APR-246 GI50 (µM ± SEM)
OACM5.1	OAC	c.743G>A	p.R248Q	DNA binding	Missense	10.7 ± 0.5
Eso26	OAC	c.742C>T	p.R248W	DNA binding	Missense	13.1 ± 3.3
FLO-1	OAC	c.830G>T	p.C277F	DNA binding	Missense	15.2 ± 1.4
OE33	OAC	c.404G>A	p.C135Y	DNA binding	Missense	40.5 ± 0.9
OANC1	OAC	c.856G>A	p.E286K	DNA binding	Missense	19.6 ± 2.5
JH-EsoAd1	OAC	c.797G>A	p.G266E	DNA binding	Missense	25.0 ± 1.1
SKGT4	OAC	c.298C>T	p.Q100*	DNA binding	Truncation	30.9 ± 3.6
OE19	OAC	c.928-929insA	p.N310fs	Tetramerisation	Frameshift	26.1 ± 4.2
OACP4C	OAC	c.574C>T	p.Q192*	DNA binding	Null	85.6 ± 7.3
TE7	OSCC	c.375G>A	Splice site	DNA binding	Null	74.4 ± 0.3
NES	Normal oesophagus	wt	wt	NA	wt	52.6 ± 1.0
MKN74	GC	c.751A>C	p.I251L	DNA binding	Missense	ND
H1299	NSCLC	Homozygous partial deletion	Null	NA	Null	73.7 ± 1.2
**Patient xenografts**						
PDX1	OAC	c.913A>T	p.K305*	Tetramerisation	Truncation	NA
PDX2	OAC	wt	wt	NA	wt	NA
PDX3	OSCC	wt	wt	NA	wt	NA
PDX4	OAC	c.796G>A	p.G266R	DNA binding	Missense	NA

Oesophageal adenocarcinoma (OAC), oesophageal squamous cell carcinoma (OSCC), gastric cancer (GC), non-small cell lung cancer (NSCLC), patient-derived xenografts (PDX), wild-type (wt), not applicable (NA), not determined (ND), growth inhibition at 50% (GI50). Although p53 mutations were identified in OACP4C and TE7 cells, these lines have been classified as p53 null as no protein was detected by western blot after loading 100 µg of whole cell lysate and probing with anti-p53 (DO-1) and (1801) antibodies.

Supplementary Table 2. PCR conditions and primer sequences for *TP53* Sanger sequencing

***TP53* exon**	**Forward (5'- 3')**	**Reverse (5'- 3')**	**Annealing Temp (°C)**	**$MgCl_2$ (25mmol L^{-1})**
**2**	GGGTTGGAAGTGTCTCATGC	CTTCCAATGGATCCACTCAC	55	No
**3**	CATGGGACTGACTTTCTGCTC	GGCAAGGGGGACTGTAGATG	55	No
**4**	CCTGGTCCTCTGACTGCTCTTTTCACCCA	GGCCAGGCATTGAAGTCTCAT	64	Yes
**5**	CAACTCTGTCTCCTTCCT	TGTCGTCTCTCCAGCCCC	55	Yes
**6**	AGAGACGACAGGGCTGGTTG	CTTAACCCCTCCTCCCAGAG	63	No
**7**	CCTCATCTTGGGCCTGTGTT	AGTGTGCAGGGTGGCAAGTG	63	No
**8**	CCTTACTGCCTCTTGCTTCT	ATAACTGCACCCTTGGTCTC	60	Yes
**9**	GGAGACCAAGGGTGCAGTTATGCCTCAG	CCCAATTGCAGGTAAAACAG	64	Yes
**10**	CATGTTGCTTTTGTACCGTCA	CAGCTGCCTTTGACCATGAA	55	No
**11**	TCATCTCTCCTCCCTGCTTC	GGGTTCAAAGACCCAAAACC	64	Yes

Supplementary Table 3. Antibodies for western blotting

Antibody	Origin	Clone	Dilutions	Source
**Anti-SLC7A11**	Rabbit	D2M7A	1:1000	Cell Signaling Technology
**Anti-NRF2**	Rabbit	D1Z9C	1:1000	Cell Signaling Technology
**Anti-p53**	Mouse	DO-1 and 1801	1:20	Hybridomas, gifts from David Lane (A*Star, Singapore)
**Anti-caspase-3**	Mouse	CPP32	1:1000	BD Biosciences
**Anti-cleaved PARP**	Rabbit	D175	1:1000	Cell Signaling Technology
**Anti-tubulin**	Mouse	B-5-1-2	1:7000	Sigma-Aldrich
**Anti-β-actin**	Mouse	C4	1:7000	MP-Biomedical
**Anti-GAPDH**	Mouse	6C5	1:7000	Abcam
**Swine anti-rabbit**	Swine	P0217	1:7000	Dako
**Goat anti-mouse**	Goat	P0447	1:7000	Dako

Supplementary Table 4. RT-PCR primer sequences

Gene	Forward (5'- 3')	Reverse (5'- 3')
SLC7A11	ATGCAGTGGCAGTGACCTTT	GGCAACAAAGATCGGAACTG
SLC3A2	AGCATCCGTGTCATTCTG	GAGCATCCTTCACCTTGG
GCLC	GGCGATGAGGTGGAATACAT	CCTGGTGTCCCTTCAATCAT
GCLM	TAATCTTGCCTCCTGCTG	CACAATGACCGAATACCG
GSS	TGCTAAAGCCCCAGAGAGAG	AGCAGGCAATTCTCAAAAGG
GSR	TCACCAAGTCCCATATAGAAATC	TGTGGCGATCAGGATGTG
GPX1	CTCTTCGAGAAGTGCGAGGT	TCGATGTCAATGGTCTGGAA
GPX4	GCACATGGTTAACCTGGACA	CTGCTTCCCGAACTGGTTAC
NRF2	CTTGGCCTCAGTGATTCTGAAGTG	CCTGAGATGGTGACAAGGGTTGTA
NQO1	CAGCTCACCGAGAGCCTAGT	GAGTGAGCCAGTACGATCAGTG
HMOX1	GGGTGATAGAAGAGGCCAAGA	AGCTCCTGCAACTCCTCAAA
PRDX1	CACTGACAAACATGGGGAAGT	TTTGCTCTTTTGGACATCAGG
KEAP1	GGGAGGTGGCCAAGCAAGAGG	TCACCTGCGTGGGCTTGTGCAG
OSGIN1	CTGCCTGTCAGGTCCGCTGC	GCGTGCTCCTTCCGGTGCTT
GAPDH	GGTGTGAACCATGAG	CCAGCAGTTTCCCGGA

Supplementary Table 5. siRNA sequences

ID	**p53** (M-003329-03)	**NRF2** (M-003755-02)	**SLC7A11** (M-007612-01)
**1**	GAGGUUGGCUCUGACUGUA	GAGAAAGAAUUGCCUGUAA	GGAAGUCUUUGGUCCAUUA
**2**	GCACAGAGGAAGAGAAUCU	CCAAAGAGCAGUUCAAUGA	GGAGUUAUGCAGCUAAUUA
**3**	GAAGAAACCACUGGAUGGA	UAAAGUGGCUGCUCAGAAU	GGGAACAACUAUAAAGAAA
**4**	GCUUCGAGAUGUUCCGAGA	UGACAGAAGUUGACAAUUA	UGACAAAUGUGGCCUACUU

Supplementary References

1. Chorley BN*, et al.* Identification of novel NRF2-regulated genes by ChIP-Seq: influence on retinoid X receptor alpha. *Nucleic Acids Res* **40**, 7416-7429 (2012).

2. Rotblat B, Grunewald TG, Leprivier G, Melino G, Knight RA. Anti-oxidative stress response genes: bioinformatic analysis of their expression and relevance in multiple cancers. *Oncotarget* **4**, 2577-2590 (2013).
